# Surgical Management of an Incidental Aortic Valve Papillary Fibroelastoma: A Case Report

**DOI:** 10.7759/cureus.108312

**Published:** 2026-05-05

**Authors:** Yassine Morjane, Oumayma Taytay, Mohamed Taha Berkane, Hicham El Malki, El Mehdi Moutaouekkil

**Affiliations:** 1 Department of Cardiovascular Surgery, Mohammed VI University Hospital Center, Oujda, MAR; 2 Faculty of Medicine and Pharmacy, Mohammed the First University, Oujda, MAR

**Keywords:** cardiac papillary fibroelastoma, cardiac tumor, case report, surgical resection, transesophageal echocardiography (tee)

## Abstract

Papillary fibroelastoma (PFE) is an uncommon benign primary cardiac tumor whose detection has increased with the widespread use of advanced cardiac imaging, particularly transesophageal echocardiography and cardiac magnetic resonance imaging. Its clinical expression is heterogeneous, ranging from incidental findings in asymptomatic individuals to serious complications related to embolic events or myocardial ischemia. Surgical excision remains the definitive treatment to eliminate this risk.

We report the case of a 69-year-old woman with a medical history of diabetes mellitus, hypertension, and coronary artery disease previously managed with percutaneous stenting of the right coronary artery and the left anterior descending artery. During routine follow-up, transesophageal echocardiography revealed an incidental mass arising from the aortic valve, specifically from the arterial aspect of the non-coronary cusp. The main differential diagnoses included thrombus, myxoma, and infective endocarditis vegetation. The patient underwent successful complete excision of the lesion under cardiopulmonary bypass, with preservation of native aortic valve function.

## Introduction

Papillary fibroelastoma (PFE) is an uncommon benign tumor of the heart, representing a small proportion of primary cardiac neoplasms and ranking third in frequency after myxomas and lipomas [1]. In earlier reports, these lesions were most often identified incidentally during cardiac surgery or postmortem examinations [2]. Over the past decades, however, the increasing use of advanced imaging modalities, particularly transesophageal echocardiography and cardiac magnetic resonance imaging, has led to more frequent detection in clinical practice [3,4].

We describe the case of a 69-year-old woman evaluated in the cardiology department during routine follow-up, in whom an aortic valve papillary fibroelastoma was identified. The patient underwent successful conservative surgical excision under cardiopulmonary bypass.

This case illustrates the variable clinical spectrum of PFE and underlines the need to consider this diagnosis when an intracardiac mass is detected, especially in patients presenting with anginal symptoms or unexplained neurological events.

## Case presentation

A 69-year-old woman with a past medical history of diabetes mellitus, hypertension, and coronary artery disease, previously managed by percutaneous stenting of the right coronary artery and the left anterior descending artery, was evaluated during routine follow-up.

**Figure 1:**
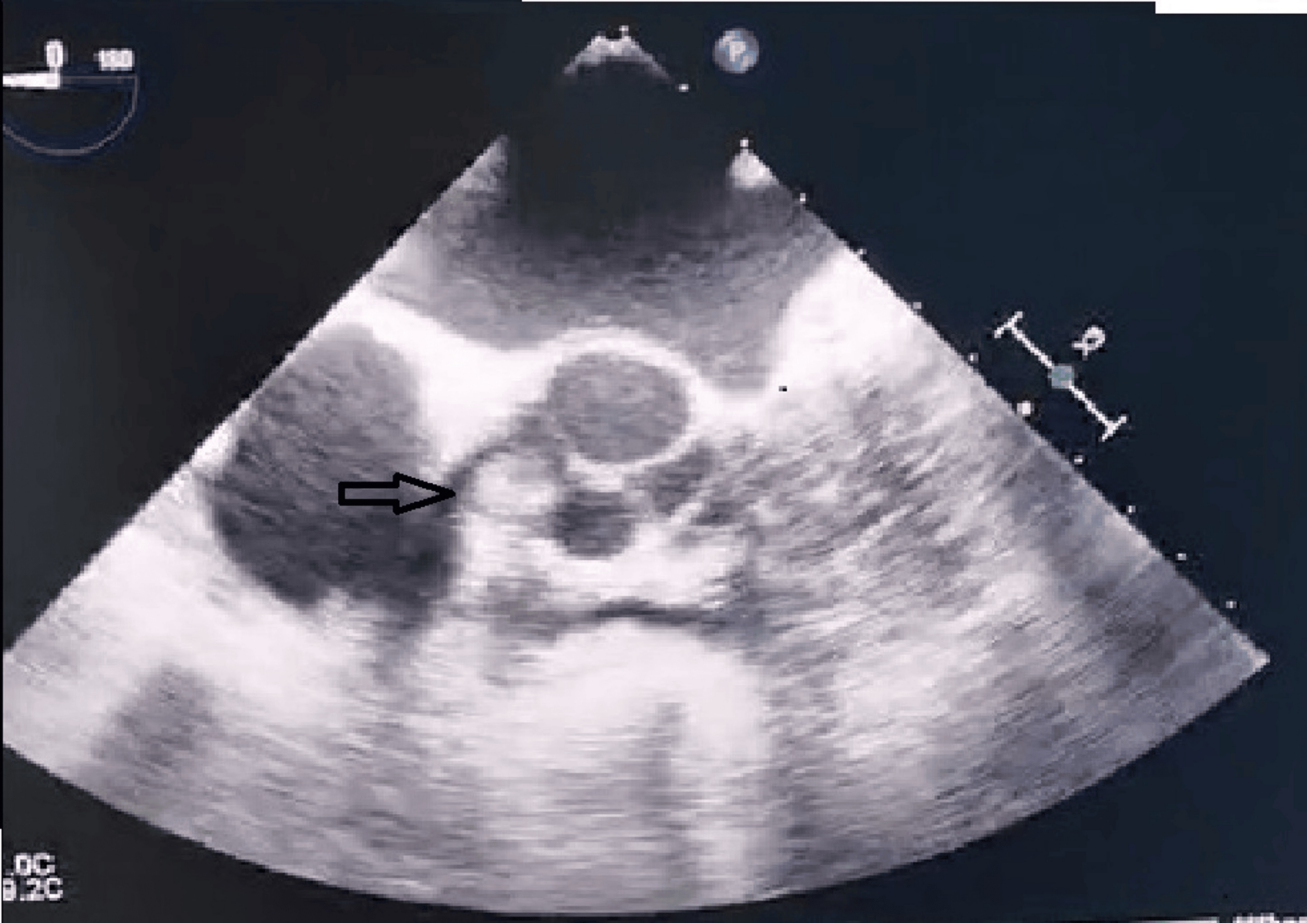
Transesophageal echocardiography (diastolic phase) demonstrating a papillary fibroelastoma arising from the aortic valve.

Transthoracic echocardiography incidentally identified a well-circumscribed, mobile, hyperechoic intracardiac mass. The patient was asymptomatic, and both physical examination and routine laboratory investigations were within normal limits. Further evaluation with transesophageal echocardiography demonstrated a pedunculated, rounded, and highly mobile supravalvular lesion measuring 12 × 8 mm. The mass was located approximately 1 cm above the aortic annulus, in close proximity to the non-coronary cusp, and exhibited regular contours (Figure 1).

**Figure 2:**
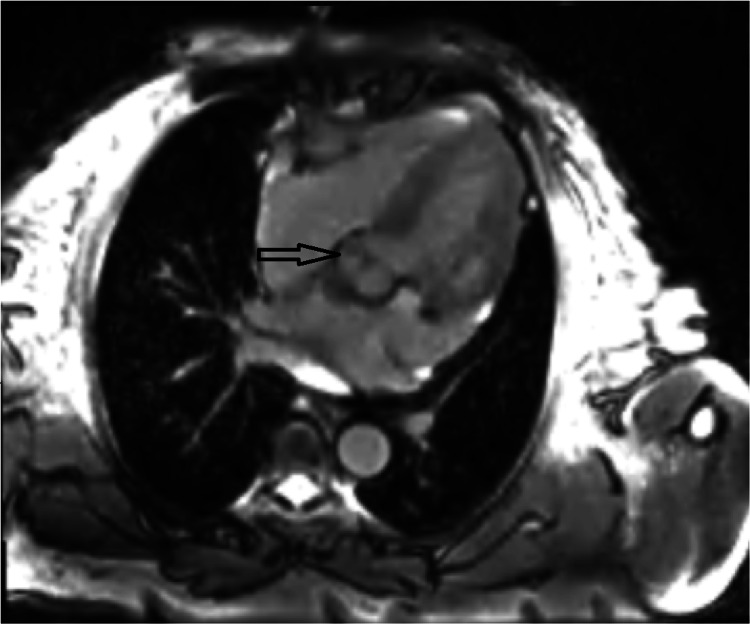
Cardiac magnetic resonance characterization of a papillary fibroelastoma.

The aortic valve was structurally normal, and no additional abnormalities were observed. To further characterize and assess the lesion, cardiac magnetic resonance imaging was performed. It demonstrated a filamentous, highly mobile mass arising from the non-coronary cusp of the aortic valve, measuring 9.5 × 2.1 mm (Figure 2).

Blood cultures were negative, and no biological signs of inflammation were present.

The differential diagnosis included thrombus, myxoma, papillary fibroelastoma, and, less likely, an inflammatory lesion. In view of the potential risk of systemic embolization, surgical intervention was indicated.

**Figure 3:**
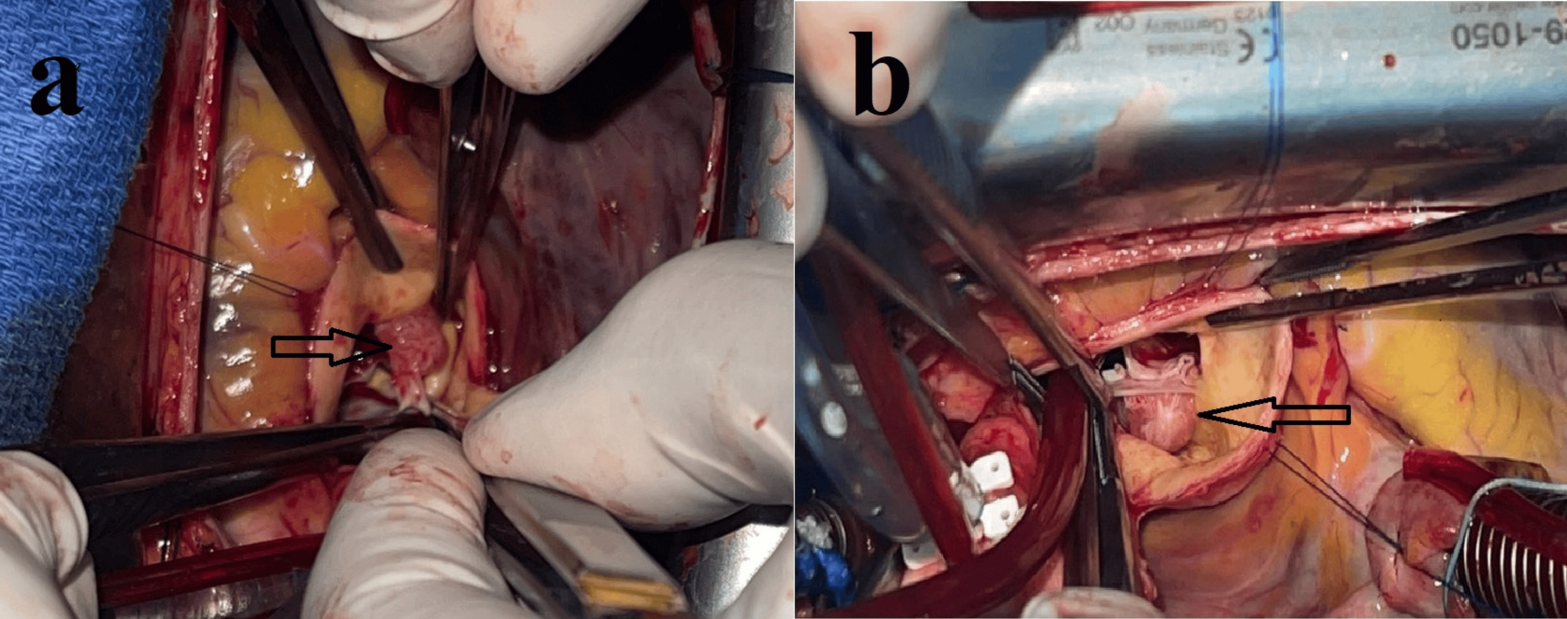
Intraoperative view showing a papillary fibroelastoma originating from the non-coronary cusp (a, b).

The patient underwent median sternotomy under standard cardiopulmonary bypass. Myocardial protection was achieved using a single dose of Custodiol® solution following aortic cross-clamping. After transverse aortotomy, intraoperative exploration revealed a small, friable, and filamentous mass attached to the arterial aspect of the noncoronary cusp, measuring approximately 8 × 6 mm (Figures 3a, 3b).

**Figure 4:**
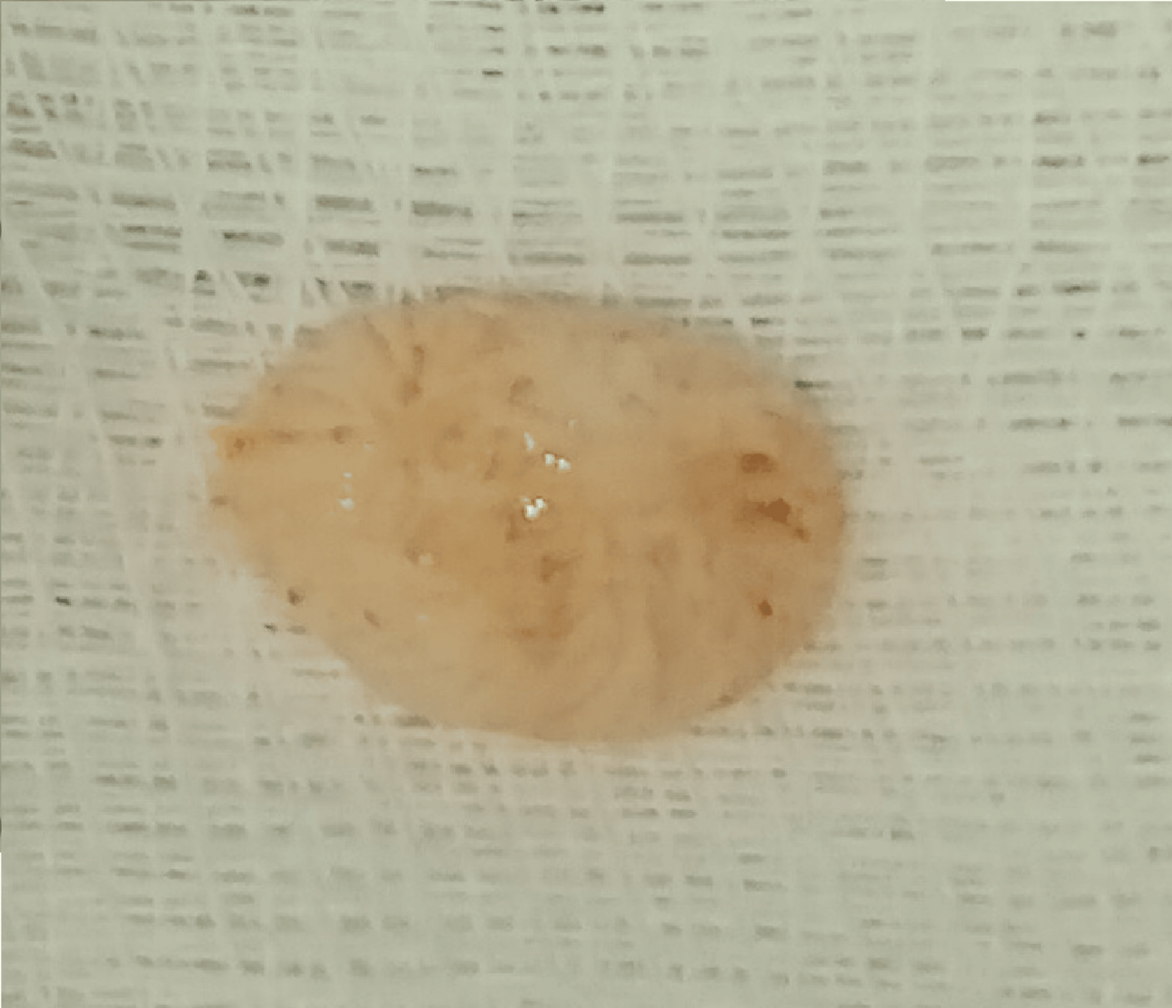
Intraoperative image after complete excision of the papillary fibroelastoma measuring 8 × 6 mm.

The aortic valve was tricuspid and functionally intact. Complete excision of the lesion was performed with preservation of the native valvular architecture (Figure 4).

Intraoperative transesophageal echocardiography confirmed the absence of residual mass and demonstrated normal valve function without regurgitation or structural damage. The patient was weaned from cardiopulmonary bypass without difficulty.

**Figure 5:**
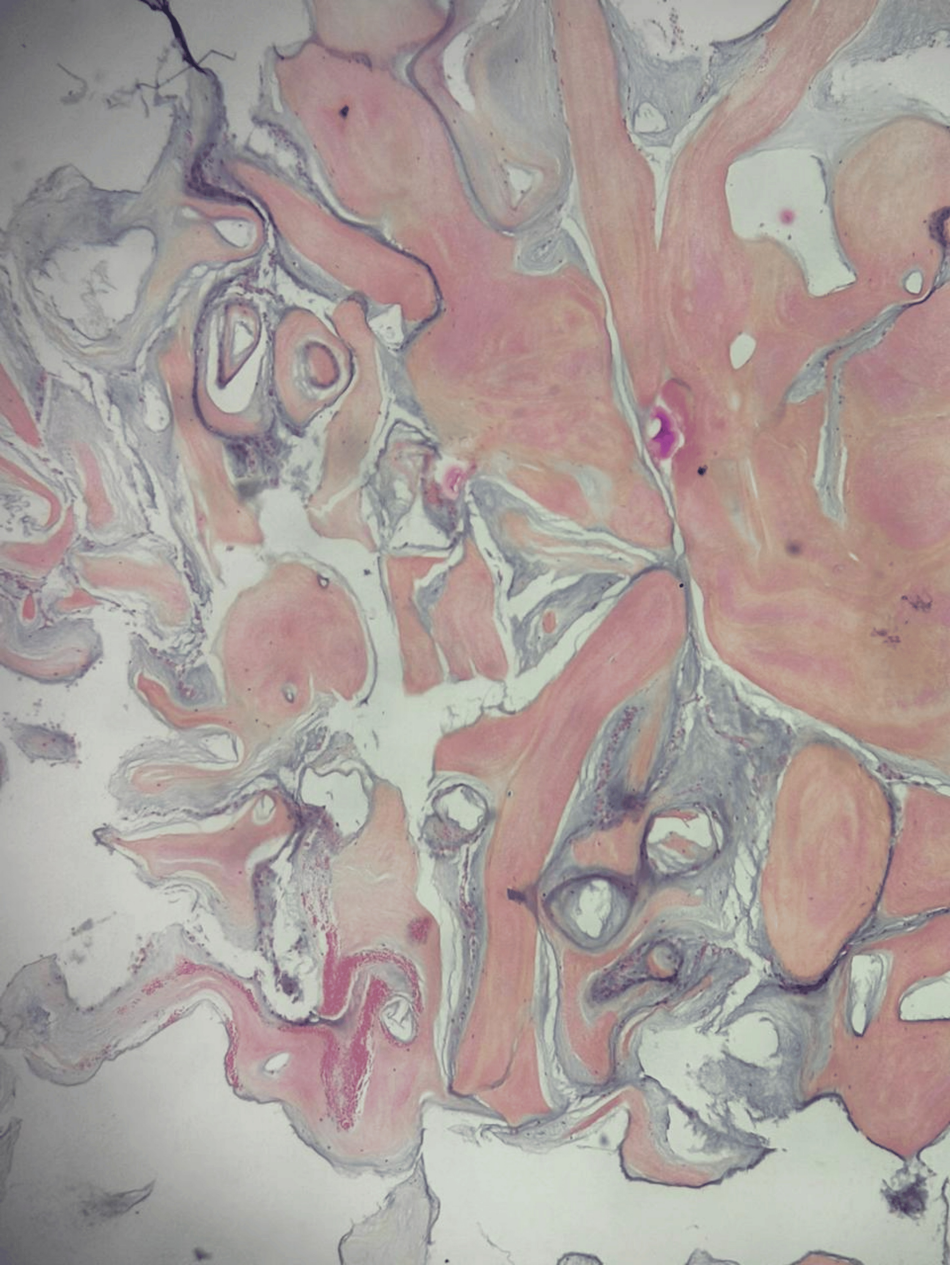
Histopathological examination revealing a papillary lesion composed of a single layer of endothelial cells lining a hyalinized and focally edematous connective tissue core, consistent with papillary fibroelastoma.

The postoperative course was uneventful. Histopathological examination revealed a papillary lesion composed of a single layer of endothelial cells lining a hyalinized and focally edematous connective tissue core, consistent with the diagnosis of papillary fibroelastoma (Figure 5).

## Discussion

Papillary fibroelastomas are rare benign tumors arising from the endocardial surface and composed of elements normally present in the endocardium, including fibrous tissue, elastic fibers, and smooth muscle cells. Morphologically, they are characterized by a small pedunculated base and multiple delicate papillary projections, classically described as resembling a sea anemone [3].

Most PFEs (approximately 85%) develop on valvular structures. The aortic valve is the most frequently affected site (29%), followed by the mitral (25%), tricuspid (17%), and pulmonary valves (13%) [3]. Although less common, non-valvular locations account for nearly 16% of cases and may involve the endocardial surfaces of cardiac chambers, papillary muscles, chordae tendineae, or even the coronary ostial intima [3-5]. Rare multifocal forms have been described, sometimes producing a “carpet-like” involvement of the left ventricular cavity [6].

The exact pathogenesis of PFEs remains a subject of debate. They have been variously interpreted as hamartomatous lesions, organized thrombi, reactive proliferations secondary to endothelial injury or infection, or iatrogenic lesions. Nevertheless, increasing evidence supports the concept that PFEs represent true neoplastic entities [3-7]. They are typically solitary lesions, although multifocal presentations are reported in less than 10% of cases [8].

Clinically, PFEs demonstrate a broad spectrum of presentation, ranging from completely asymptomatic incidental findings to life-threatening embolic events. Reported complications include transient ischemic attacks, ischemic stroke, myocardial infarction, and systemic embolization. Less commonly, pulmonary embolism, syncope, arrhythmias such as ventricular fibrillation, heart failure, and sudden cardiac death have also been described [3-5].

In the present case, the patient’s chest pain with angina-like characteristics may be explained by intermittent obstruction of blood flow at the level of the aortic root, likely related to the tumor’s mobility and its position during the cardiac cycle.

Diagnosis is primarily based on echocardiographic evaluation. Transthoracic echocardiography is often the first-line modality, while transesophageal echocardiography provides superior spatial resolution and a more precise assessment of tumor attachment, mobility, and relation to adjacent structures [9]. Cross-sectional imaging techniques such as cardiac magnetic resonance imaging and multidetector computed tomography may provide complementary information, particularly when echocardiographic findings are inconclusive [10,11]. Spiral CT and MRI are also useful alternatives when transesophageal echocardiography is contraindicated [12].

Echocardiographically, PFEs typically appear as small, highly mobile, pedunculated masses with a characteristic, frond-like or filamentous architecture. This appearance, although suggestive, is not specific. The main differential diagnoses include intracardiac thrombus; benign cardiac tumors such as myxoma or lipoma; and infective endocarditis vegetations, particularly in the presence of systemic infection [3,8].

Despite their histologically benign nature, PFEs are clinically relevant due to their marked embolic potential, which is related to their mobility and friable papillary fronds [3-13]. For this reason, surgical excision is generally recommended once the diagnosis is established, even in asymptomatic patients, particularly when the lesion is mobile or located on the left side of the heart [3,5,13-15].

Surgical management is usually straightforward, with a high rate of valve preservation. However, in cases where valvular integrity is compromised, repair or replacement may be necessary [5,13,15,16]. Intraoperative transesophageal echocardiography following aortic unclamping plays a crucial role in confirming complete resection and assessing immediate valvular function.

## Conclusions

Papillary fibroelastoma is a benign cardiac tumor, but its location and high mobility are associated with a clinically relevant risk of severe thromboembolic events. When this diagnosis is suspected, transesophageal echocardiography remains the key imaging modality for accurate identification and characterization of the lesion. Early surgical excision, often preceded by appropriate perioperative anticoagulation, represents the optimal therapeutic strategy, effectively reducing the risk of embolic and neurological complications and ensuring an excellent prognosis.
